# Whole genome sequence of denitrifying bacterium *Stutzerimonas stutzeri* strain NGHE31, collected from an eutrophic wetland in Sunamganj, Bangladesh, following the 2017 flash floods

**DOI:** 10.1128/mra.00001-24

**Published:** 2024-02-23

**Authors:** Aura Rahman, Sumaiya Akter, Abdus Sadique, Jahidul Alam, Shoheli Asrafie, Anika Tabassum, Sakib Abrar Hossain, Mohammad Moshiur Rahman, Md. Baki Billah, Tahrima Saiha Huq, Kabir M. Uddin, Md. Mahbubur Rahman, Ayesha Sharmin, Md. Mainul Hossain, Sayad Md. Didarul Alam, Maqsud Hossain

**Affiliations:** 1 NSU Genome Research Institute (NGRI), North South University, Dhaka, Bangladesh; 2 Department of Biochemistry and Microbiology, School of Health and Life Sciences, North South University, Dhaka, Bangladesh; 3 Department of Environmental Science and Management, School of Health and Life Sciences, North South University, Dhaka, Bangladesh; 4 Department of Zoology, Jahangirnagar University, Savar, Dhaka, Bangladesh; 5 Department of Chemistry, Bangladesh University of Engineering & Technology, Dhaka, Bangladesh; University of Southern California, Los Angeles, California, USA

**Keywords:** *Stutzerimonas stutzeri*, genome sequence

## Abstract

Here, we report the whole genome sequence of *Stutzerimonas stutzeri* strain NGHE31, isolated from Dekhar Haor, following the 2017 flash flood that resulted in mass die-offs of local wildlife. The predicted genome size is 4,434,670 bp, with 63.97% GC content, 4,035 coding sequences, 3 rRNAs, and 50 tRNAs.

## ANNOUNCEMENT

*Stutzerimonas stutzeri* is a Gram-negative, facultative anaerobic bacterium belonging to the Gammaproteobacteria that has been frequently reported to coexist with cyanobacterial species in eutrophic ecosystems (1–3). *S. stutzeri* is an important natural denitrifier, and studies have also shown its promising applicability in bioremediation, pollutant degradation, and wastewater treatment, owing to its rich repertoire of genes involved in the metabolism of organic and toxic compounds (4–6). The 2017 flashfloods in Dekhar Haor (lies between latitude 24°34′N to 25°12′N and longitude 90°56′E to 91°49′E), one of the most important wetland ecosystems in Sunamganj, Bangladesh, saw a large number of deaths in wildlife, including fishes, migratory birds and cattle.

Following the floods in March 2017, 500 mL of sub-surface (ca. 0.5 m depth) water sample was collected into a sterile plastic bottle (Nalgene, USA) from Dekhar Haor. The sample was transported in an insulated plastic box and processed within 18 h of collection. The water was filtered through a 0.22 µm, 47 mm polycarbonate membrane filter (Millipore Corp., Bedford, MA, USA), and the filter biomass including bacteria was used to inoculate the BG-11 medium. From the culture, 100 µL was spread on BG-11 agar plate and incubated at room temperature for 1 week exposed to sunlight for isolates. Identification was confirmed by microscopic and biochemical analysis according to the method described previously (7). A single colony was then used to inoculate the BG-11 medium and incubated at room temperature for 48 hours under a natural day/night cycle. From the cultured broth, 2 mL was pelleted to extract microbial genomic DNA using Qiagen DNeasy PowerBiofilm Kit. DNA quality and quantity were checked using NanoDrop One (Thermo Scientific, USA). The sequencing library was generated using Illumina DNA Prep workflow and Nextera DNA CD indexes. The library was sequenced on Illumina MiSeq using 250-cycle paired-end chemistry, following which sequence read files were generated using the built-in FASTQ Generation tool (version 1.0.0) on Illumina’s BaseSpace Sequencing Hub, with default settings and adaptor trimming enabled. The sequencing produced 608,632 paired-end reads of ~250 bp size. The initial quality of the reads was checked using FastQC (http://www.bioinformatics.babraham.ac.uk/projects/fastqc), and quality trimming was done by Sickle version 0.991 (https://github.com/najoshi/sickle). The reads were then assembled using SPAdes version 3.15.4 with a coverage cutoff of 20× (8), following which contigs smaller than 200 nt were filtered out. Default parameters were used for all software unless otherwise specified. The assembly produced 40 contigs with a total sequence length of 4,434,670 bp, GC content of 63.97%, and *N*_50_ value of 292,971 bp. The 16s rRNA gene sequence was then compared using the online NCBI BLAST server and FastANI, which revealed 100% and 97.68% similarity, respectively, with RefSeq reference genome NZ_AP024722.1, suggesting the species to be *S. stutzeri*. Annotation with NCBI Prokaryotic Genome Annotation Pipeline version 6.1 (9) revealed the genome to contain 4,035 coding DNA sequences, 50 tRNAs, and 3 rRNAs. Further annotation was performed using RASTtk (10), and it revealed NGHE31 to contain 33 genes for denitrification and 7 genes for selenoprotein metabolism.

## Data Availability

The sequence reads generated in this study have been submitted to the Sequence Read Archive (SRA) under the accession number SRR22947188. The assembled genome has been deposited to NCBI GenBank under the accession number JANAJI000000000.1 and the BioProject accession number PRJNA855290. The RASTtk annotation files have been published on Figshare (DOI: https://doi.org/10.6084/m9.figshare.25200878.v2).
